# *rac*-Methyl (1*R*,3′*S*)-1′,1′′-dimethyl-2,2′′-dioxo-2*H*-dispiro­[acenaphthyl­ene-1,2′-pyrrolidine-3′,3′′-indoline]-4′-carboxyl­ate

**DOI:** 10.1107/S1600536813000470

**Published:** 2013-01-12

**Authors:** G. Ganesh, Panneer Selvam Yuvaraj, Chinthalapuri Divakara, Boreddy S. R. Reddy, A. SubbiahPandi

**Affiliations:** a Department of Physics, S.M.K. Fomra Institute of Technology, Thaiyur, Chennai 603 103, India; b Industrial Chemistry Laboratory, Central Leather Research Institute, Adyar, Chennai 600 020, India; c Department of Physics, Presidency College (Autonomous), Chennai 600 005, India

## Abstract

In the title compound, C_26_H_22_N_2_O_4_, the pyrrolidine ring adopts a twisted conformation and the other five-membered rings adopt envelope conformations with the spiro C atoms as the flap atoms. The naphthalene ring system of the dihydro­acenaphthyl­ene group forms dihedral angles of 89.2 (9) and 75.5 (6)° with the pyrrolidine and indole rings, respectively. The pyrrolidine ring makes a dihedral angle of 80.1 (9)° with the indole ring. In the crystal, mol­ecules are linked by weak C—H⋯O hydrogen bonds, forming chains along the *b-*axis direction.

## Related literature

For the biological activity of naphthalene derivatives, see: Wiltz *et al.* (1998 ▶); Wright *et al.* (2000 ▶); Varma *et al.* (1994 ▶). For a related structure, see: Wei *et al.* (2012 ▶). For ring conformations, see: Cremer & Pople (1975 ▶).
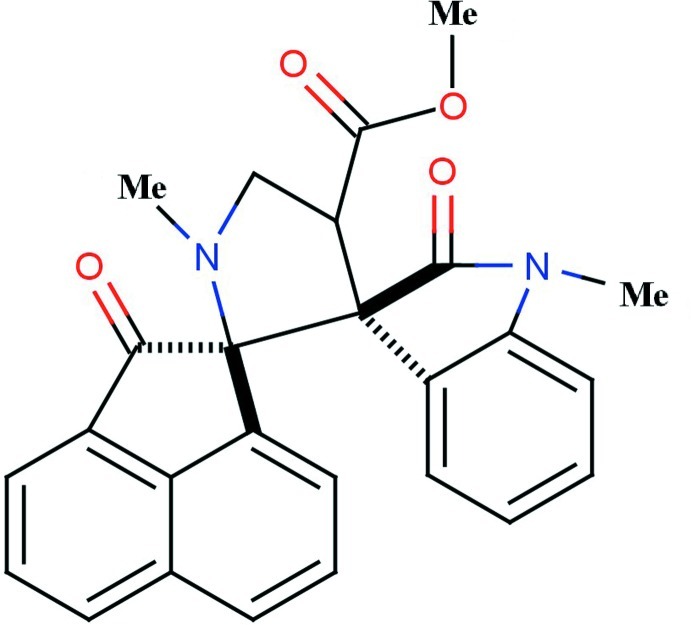


## Experimental

### 

#### Crystal data

C_26_H_22_N_2_O_4_
*M* *_r_* = 426.46
Monoclinic, 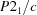

*a* = 15.4839 (4) Å
*b* = 9.5832 (2) Å
*c* = 15.6375 (4) Å
β = 115.184 (1)°
*V* = 2099.81 (9) Å^3^
*Z* = 4
Mo *K*α radiation
μ = 0.09 mm^−1^
*T* = 293 K
0.25 × 0.22 × 0.19 mm

#### Data collection

Bruker APEXII CCD area-detector diffractometer
Absorption correction: multi-scan (*SADABS*; Bruker, 2008 ▶) *T*_min_ = 0.977, *T*_max_ = 0.983
19264 measured reflections
4057 independent reflections
3017 reflections with *I* > 2σ(*I*)
*R*_int_ = 0.031

#### Refinement

*R*[*F* ^2^ > 2σ(*F* ^2^)] = 0.038
*wR*(*F* ^2^) = 0.103
*S* = 1.03
4057 reflections
292 parameters
H-atom parameters constrained
Δρ_max_ = 0.17 e Å^−3^
Δρ_min_ = −0.17 e Å^−3^

### 

Data collection: *APEX2* (Bruker, 2008 ▶); cell refinement: *SAINT* (Bruker, 2008 ▶); data reduction: *SAINT*; program(s) used to solve structure: *SHELXS97* (Sheldrick, 2008 ▶); program(s) used to refine structure: *SHELXL97* (Sheldrick, 2008 ▶); molecular graphics: *ORTEP-3* (Farrugia, 2012 ▶); software used to prepare material for publication: *SHELXL97* and *PLATON* (Spek, 2009 ▶).

## Supplementary Material

Crystal structure: contains datablock(s) global, I. DOI: 10.1107/S1600536813000470/lx2278sup1.cif

Structure factors: contains datablock(s) I. DOI: 10.1107/S1600536813000470/lx2278Isup2.hkl

Additional supplementary materials:  crystallographic information; 3D view; checkCIF report

## Figures and Tables

**Table 1:** Hydrogen-bond geometry (Å, °)

*D*—H⋯*A*	*D*—H	H⋯*A*	*D*⋯*A*	*D*—H⋯*A*
C10—H10⋯O2^i^	0.93	2.60	3.268 (2)	130

Symmetry code: (i) 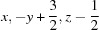.

**Fig. 1.**
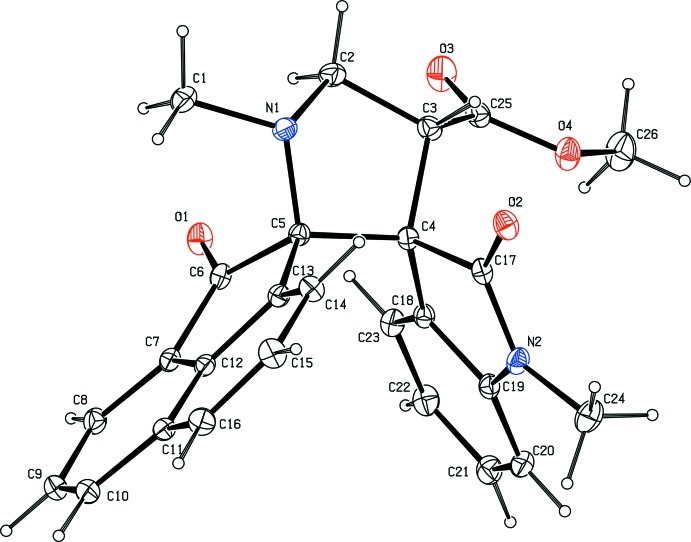
The molecular structure of the title compound with the atom numbering scheme. Displacement ellipsoids are drawn at the 30% probability level. H atoms are presented as small spheres of arbitrary radius.

## supplementary crystallographic information

### Comment

Naphthalene derivatives have manifested applications in many fields, for example, as a colorant, explosive, disinfectant, insecticide and plant hormone auxin. Naphthalene is believed to play a role in the chemical defence against biological enemies (Wiltz *et al.*, 1998; Wright *et al.*, 2000). It may be produced by metabolic processes in termites or by associated microorganisms which inhabit, e.g., the termite guts (Varma *et al.*, 1994). In view of these importance and continuation of our work on the crystal structure analyis of napthalene derivatives, the crystal structure of the title compound has been carried out and the results are presented here.

X–Ray analysis confirms the molecular structure and atom connectivity as illustrated in Fig. 1. The geometry of acenaphthylene and pyrrolidine ring systems are comparable with the related structure [Wei *et al.* (2012)]. The sum of the angles at N1 [338.2 (1)°] and N2 [359.4 (1)°] of the pyrrolidine rings are in accordance with *sp*^3^ and *sp*^2^ hybridizations. The naphthalene ring system of the dihydroacenaphthylene group [C7–C16] forms dihedral angles of 89.2 (8) and 75.5 (5)° with the central pyrrolidine ring [N1/C2–C5] and the indole ring [N2/C4/C17–C23], respectively. It clearly shows that the naphthalene ring system of the dihydroacenaphthylene group attached to the central pyrrolidine ring are almost perpendicular to each other. Also the dihedral angle between the central pyrrolidine and the indole ring forms a a dihedral angle of 80.1 (8)°.

The central pyrrolidine ring adopts twisted conformations on N1 and C2 atoms with the pukering parameter of q_2_ = 0.3942 (2) Å, φ = 13.72 (3)° (Cremer & Pople, 1975). The pyrrolidine ring [N2/C4/C17–C19] in the indole group adopts envelope conformations, q_2_ = 0.0889 (2) Å and φ = 219.39 (1)°, and with atom C17 deviating -0.0565 (2) Å from the least–squares plane passing through the remaining four atoms (N2/C19/C18/C4) of that ring. In the crystal the molecules are linked by weak intermolecular C—H···O hydrogen bonds (Table 1), forming one-dimensional chains along the *b*–axis.

### Experimental

To a mixture of 1eq of (*E*)–methyl 2–(1–methyl–2–oxoindolin –3–ylidene) acetate, 1eq of isatin and 1.5eq of acenaphthylene–1,2 –dione were dissolved in acetonitrile. This reaction mixture refluxed at 80°C for 8 hours. The reaction mixture was monitored for completion by thin layar chromatography. Upon completion, the reaction mixture was extracted with ethyl acetate and water. The product was dried and purified by coloumn chromatography using ethyl acetate and hexane (1:9) as an elutent to affored pure Dispiro oxindole. Yield (78%). Single crystals suitable for X–ray diffraction were obtained by slow evaporation of a solution of the title compound in ethyl acetate at room temperature.

### Refinement

All H atoms were fixed geometrically and allowed to ride on their parent C atoms, with C—H distances fixed in the range 0.93–0.97 Å with *U*_iso_(H) = 1.5*U*_eq_(C) for methyl H 1.2*U*_eq_(C) for other H atoms. The positions of methyl hydrogens were optimized rotationally.

### Crystal data

**Table:**

C_26_H_22_N_2_O_4_	*F*(000) = 896
*M**_r_* = 426.46	*D*_x_ = 1.349 Mg m^−^^3^
Monoclinic, *P*2_1_/*c*	Mo *K*α radiation, λ = 0.71073 Å
Hall symbol: -P 2ybc	Cell parameters from 4057 reflections
*a* = 15.4839 (4) Å	θ = 1.5–25.8°
*b* = 9.5832 (2) Å	µ = 0.09 mm^−^^1^
*c* = 15.6375 (4) Å	*T* = 293 K
β = 115.184 (1)°	Block, colourless
*V* = 2099.81 (9) Å^3^	0.25 × 0.22 × 0.19 mm
*Z* = 4	

### Data collection

**Table:**

Bruker APEXII CCD area-detector diffractometer	4057 independent reflections
Radiation source: fine-focus sealed tube	3017 reflections with *I* > 2σ(*I*)
Graphite monochromator	*R*_int_ = 0.031
ω and φ scans	θ_max_ = 25.8°, θ_min_ = 2.6°
Absorption correction: multi-scan (*SADABS*; Bruker, 2008)	*h* = −18→18
*T*_min_ = 0.977, *T*_max_ = 0.983	*k* = −11→11
19264 measured reflections	*l* = −19→18

### Refinement

**Table:**

Refinement on *F*^2^	Primary atom site location: structure-invariant direct methods
Least-squares matrix: full	Secondary atom site location: difference Fourier map
*R*[*F*^2^ > 2σ(*F*^2^)] = 0.038	Hydrogen site location: difference Fourier map
*wR*(*F*^2^) = 0.103	H-atom parameters constrained
*S* = 1.03	*w* = 1/[σ^2^(*F*_o_^2^) + (0.0432*P*)^2^ + 0.5222*P*] where *P* = (*F*_o_^2^ + 2*F*_c_^2^)/3
4057 reflections	(Δ/σ)_max_ < 0.001
292 parameters	Δρ_max_ = 0.17 e Å^−^^3^
0 restraints	Δρ_min_ = −0.17 e Å^−^^3^

### Special details

**Table:**

Geometry. All esds (except the esd in the dihedral angle between two l.s. planes) are estimated using the full covariance matrix. The cell esds are taken into account individually in the estimation of esds in distances, angles and torsion angles; correlations between esds in cell parameters are only used when they are defined by crystal symmetry. An approximate (isotropic) treatment of cell esds is used for estimating esds involving l.s. planes.
Refinement. Refinement of F^2^ against ALL reflections. The weighted R-factor wR and goodness of fit S are based on F^2^, conventional R-factors R are based on F, with F set to zero for negative F^2^. The threshold expression of F^2^ > 2sigma(F^2^) is used only for calculating R-factors(gt) etc. and is not relevant to the choice of reflections for refinement. R-factors based on F^2^ are statistically about twice as large as those based on F, and R- factors based on ALL data will be even larger.

### Fractional atomic coordinates and isotropic or equivalent isotropic displacement parameters (Å^2^)

**Table:**

	*x*	*y*	*z*	*U*_iso_*/*U*_eq_	
O1	0.69067 (8)	0.37535 (12)	0.28269 (9)	0.0565 (3)	
O2	0.87808 (9)	0.87118 (12)	0.39458 (9)	0.0552 (3)	
O3	0.64333 (11)	0.58931 (15)	0.48841 (11)	0.0762 (4)	
O4	0.65285 (10)	0.81185 (13)	0.45236 (10)	0.0648 (4)	
N1	0.87057 (9)	0.51097 (14)	0.41944 (9)	0.0456 (3)	
N2	0.73670 (10)	0.90053 (13)	0.26438 (10)	0.0490 (3)	
C1	0.91612 (13)	0.37648 (19)	0.42302 (14)	0.0590 (5)	
H1A	0.8683	0.3050	0.3999	0.088*	
H1B	0.9596	0.3560	0.4871	0.088*	
H1C	0.9505	0.3800	0.3844	0.088*	
C2	0.81537 (13)	0.51929 (18)	0.47465 (12)	0.0537 (4)	
H2A	0.8562	0.5123	0.5418	0.064*	
H2B	0.7673	0.4465	0.4568	0.064*	
C3	0.77008 (12)	0.66228 (17)	0.44928 (11)	0.0468 (4)	
H3	0.8174	0.7303	0.4890	0.056*	
C4	0.75428 (11)	0.68832 (15)	0.34492 (11)	0.0385 (3)	
C5	0.81077 (10)	0.56359 (15)	0.32472 (10)	0.0377 (3)	
C6	0.74144 (11)	0.44844 (15)	0.25997 (12)	0.0410 (4)	
C7	0.75797 (10)	0.43438 (15)	0.17441 (11)	0.0399 (4)	
C8	0.71903 (12)	0.34921 (17)	0.09679 (12)	0.0503 (4)	
H8	0.6677	0.2913	0.0878	0.060*	
C9	0.75897 (13)	0.35191 (18)	0.03135 (12)	0.0566 (5)	
H9	0.7321	0.2965	−0.0225	0.068*	
C10	0.83614 (14)	0.43324 (18)	0.04395 (12)	0.0544 (4)	
H10	0.8613	0.4304	−0.0004	0.065*	
C11	0.87793 (12)	0.52114 (16)	0.12320 (11)	0.0437 (4)	
C12	0.83529 (10)	0.52056 (15)	0.18635 (10)	0.0375 (3)	
C13	0.86999 (10)	0.59774 (15)	0.27095 (11)	0.0377 (3)	
C14	0.95079 (11)	0.67546 (17)	0.29344 (12)	0.0470 (4)	
H14	0.9769	0.7255	0.3497	0.056*	
C15	0.99391 (13)	0.67866 (19)	0.23017 (13)	0.0550 (5)	
H15	1.0482	0.7331	0.2454	0.066*	
C16	0.95951 (13)	0.60548 (19)	0.14783 (13)	0.0545 (4)	
H16	0.9898	0.6111	0.1078	0.065*	
C17	0.79970 (12)	0.82915 (16)	0.33990 (12)	0.0430 (4)	
C18	0.65287 (11)	0.70826 (15)	0.27105 (11)	0.0394 (4)	
C19	0.64718 (11)	0.83487 (16)	0.22577 (11)	0.0429 (4)	
C20	0.56296 (13)	0.88569 (19)	0.15782 (13)	0.0555 (5)	
H20	0.5609	0.9701	0.1276	0.067*	
C21	0.48165 (13)	0.8071 (2)	0.13602 (14)	0.0601 (5)	
H21	0.4237	0.8390	0.0902	0.072*	
C22	0.48497 (12)	0.6824 (2)	0.18089 (14)	0.0584 (5)	
H22	0.4292	0.6317	0.1657	0.070*	
C23	0.57069 (11)	0.63162 (17)	0.24860 (12)	0.0489 (4)	
H23	0.5727	0.5470	0.2785	0.059*	
C24	0.75568 (16)	1.03779 (19)	0.23647 (17)	0.0747 (6)	
H24A	0.7189	1.1066	0.2511	0.112*	
H24B	0.7383	1.0385	0.1698	0.112*	
H24C	0.8224	1.0589	0.2701	0.112*	
C25	0.68278 (13)	0.68033 (18)	0.46619 (12)	0.0511 (4)	
C26	0.56590 (17)	0.8428 (3)	0.4604 (2)	0.0891 (8)	
H26A	0.5144	0.7906	0.4139	0.134*	
H26B	0.5525	0.9408	0.4503	0.134*	
H26C	0.5727	0.8178	0.5224	0.134*	

### Atomic displacement parameters (Å^2^)

**Table:**

	*U*^11^	*U*^22^	*U*^33^	*U*^12^	*U*^13^	*U*^23^
O1	0.0570 (7)	0.0447 (7)	0.0803 (9)	−0.0125 (6)	0.0413 (7)	−0.0052 (6)
O2	0.0555 (7)	0.0507 (7)	0.0624 (8)	−0.0152 (6)	0.0281 (6)	−0.0144 (6)
O3	0.0945 (10)	0.0629 (8)	0.1036 (11)	−0.0088 (8)	0.0734 (10)	0.0024 (8)
O4	0.0775 (9)	0.0521 (7)	0.0881 (10)	0.0029 (6)	0.0577 (8)	−0.0005 (7)
N1	0.0475 (8)	0.0464 (8)	0.0437 (8)	0.0061 (6)	0.0202 (6)	0.0065 (6)
N2	0.0579 (9)	0.0333 (7)	0.0603 (9)	−0.0026 (6)	0.0295 (7)	0.0028 (6)
C1	0.0604 (11)	0.0532 (11)	0.0598 (11)	0.0149 (9)	0.0223 (9)	0.0104 (9)
C2	0.0652 (11)	0.0552 (10)	0.0481 (10)	0.0046 (9)	0.0313 (9)	0.0099 (8)
C3	0.0543 (10)	0.0472 (9)	0.0452 (9)	−0.0055 (8)	0.0272 (8)	−0.0025 (7)
C4	0.0422 (8)	0.0358 (8)	0.0422 (8)	−0.0021 (6)	0.0225 (7)	−0.0013 (7)
C5	0.0387 (8)	0.0352 (8)	0.0410 (8)	−0.0005 (6)	0.0188 (7)	0.0005 (6)
C6	0.0364 (8)	0.0335 (8)	0.0546 (10)	0.0022 (6)	0.0207 (7)	0.0012 (7)
C7	0.0392 (8)	0.0320 (8)	0.0448 (9)	0.0037 (6)	0.0144 (7)	0.0016 (7)
C8	0.0502 (10)	0.0387 (9)	0.0521 (10)	−0.0008 (7)	0.0121 (8)	−0.0043 (8)
C9	0.0714 (12)	0.0467 (10)	0.0434 (10)	0.0045 (9)	0.0164 (9)	−0.0074 (8)
C10	0.0735 (12)	0.0481 (10)	0.0468 (10)	0.0071 (9)	0.0308 (9)	0.0015 (8)
C11	0.0523 (10)	0.0399 (8)	0.0421 (9)	0.0067 (7)	0.0231 (8)	0.0047 (7)
C12	0.0379 (8)	0.0331 (7)	0.0397 (8)	0.0048 (6)	0.0148 (7)	0.0037 (6)
C13	0.0362 (8)	0.0369 (8)	0.0411 (8)	0.0011 (6)	0.0176 (7)	0.0010 (7)
C14	0.0439 (9)	0.0511 (9)	0.0485 (9)	−0.0084 (7)	0.0219 (8)	−0.0058 (8)
C15	0.0484 (10)	0.0592 (11)	0.0664 (12)	−0.0132 (8)	0.0332 (9)	−0.0058 (9)
C16	0.0595 (11)	0.0573 (11)	0.0617 (11)	−0.0001 (9)	0.0402 (10)	0.0039 (9)
C17	0.0493 (9)	0.0376 (8)	0.0502 (9)	−0.0058 (7)	0.0290 (8)	−0.0091 (7)
C18	0.0411 (8)	0.0375 (8)	0.0454 (9)	0.0009 (6)	0.0241 (7)	−0.0023 (7)
C19	0.0502 (9)	0.0367 (8)	0.0483 (9)	0.0029 (7)	0.0273 (8)	−0.0026 (7)
C20	0.0653 (12)	0.0455 (10)	0.0572 (11)	0.0148 (9)	0.0274 (10)	0.0066 (8)
C21	0.0496 (10)	0.0622 (12)	0.0623 (12)	0.0147 (9)	0.0177 (9)	−0.0045 (10)
C22	0.0416 (10)	0.0616 (11)	0.0709 (12)	0.0012 (8)	0.0230 (9)	−0.0092 (10)
C23	0.0457 (9)	0.0454 (9)	0.0608 (11)	−0.0004 (7)	0.0277 (8)	−0.0006 (8)
C24	0.0924 (16)	0.0405 (10)	0.0951 (16)	−0.0103 (10)	0.0438 (13)	0.0119 (10)
C25	0.0649 (11)	0.0489 (10)	0.0522 (10)	−0.0087 (8)	0.0371 (9)	−0.0056 (8)
C26	0.0934 (17)	0.0816 (16)	0.127 (2)	0.0184 (13)	0.0803 (17)	0.0055 (14)

### Geometric parameters (Å, º)

**Table:**

O1—C6	1.2135 (18)	C9—C10	1.369 (3)
O2—C17	1.2178 (19)	C9—H9	0.9300
O3—C25	1.198 (2)	C10—C11	1.408 (2)
O4—C25	1.328 (2)	C10—H10	0.9300
O4—C26	1.436 (2)	C11—C12	1.402 (2)
N1—C2	1.453 (2)	C11—C16	1.408 (2)
N1—C1	1.459 (2)	C12—C13	1.407 (2)
N1—C5	1.4622 (19)	C13—C14	1.367 (2)
N2—C17	1.354 (2)	C14—C15	1.410 (2)
N2—C19	1.403 (2)	C14—H14	0.9300
N2—C24	1.455 (2)	C15—C16	1.360 (3)
C1—H1A	0.9600	C15—H15	0.9300
C1—H1B	0.9600	C16—H16	0.9300
C1—H1C	0.9600	C18—C23	1.379 (2)
C2—C3	1.513 (2)	C18—C19	1.389 (2)
C2—H2A	0.9700	C19—C20	1.374 (2)
C2—H2B	0.9700	C20—C21	1.379 (3)
C3—C25	1.494 (2)	C20—H20	0.9300
C3—C4	1.564 (2)	C21—C22	1.376 (3)
C3—H3	0.9800	C21—H21	0.9300
C4—C18	1.514 (2)	C22—C23	1.387 (2)
C4—C17	1.539 (2)	C22—H22	0.9300
C4—C5	1.590 (2)	C23—H23	0.9300
C5—C13	1.520 (2)	C24—H24A	0.9600
C5—C6	1.572 (2)	C24—H24B	0.9600
C6—C7	1.471 (2)	C24—H24C	0.9600
C7—C8	1.371 (2)	C26—H26A	0.9600
C7—C12	1.400 (2)	C26—H26B	0.9600
C8—C9	1.403 (2)	C26—H26C	0.9600
C8—H8	0.9300		
			
C25—O4—C26	117.20 (15)	C12—C11—C16	116.49 (15)
C2—N1—C1	115.01 (14)	C10—C11—C16	127.37 (16)
C2—N1—C5	107.70 (12)	C7—C12—C11	122.73 (14)
C1—N1—C5	115.53 (13)	C7—C12—C13	113.46 (13)
C17—N2—C19	111.21 (13)	C11—C12—C13	123.65 (14)
C17—N2—C24	123.77 (15)	C14—C13—C12	118.01 (14)
C19—N2—C24	124.40 (15)	C14—C13—C5	132.55 (14)
N1—C1—H1A	109.5	C12—C13—C5	109.14 (12)
N1—C1—H1B	109.5	C13—C14—C15	119.08 (15)
H1A—C1—H1B	109.5	C13—C14—H14	120.5
N1—C1—H1C	109.5	C15—C14—H14	120.5
H1A—C1—H1C	109.5	C16—C15—C14	122.79 (16)
H1B—C1—H1C	109.5	C16—C15—H15	118.6
N1—C2—C3	102.59 (13)	C14—C15—H15	118.6
N1—C2—H2A	111.2	C15—C16—C11	119.94 (15)
C3—C2—H2A	111.2	C15—C16—H16	120.0
N1—C2—H2B	111.2	C11—C16—H16	120.0
C3—C2—H2B	111.2	O2—C17—N2	125.31 (15)
H2A—C2—H2B	109.2	O2—C17—C4	126.49 (15)
C25—C3—C2	114.31 (14)	N2—C17—C4	108.19 (13)
C25—C3—C4	114.51 (14)	C23—C18—C19	118.95 (15)
C2—C3—C4	105.51 (13)	C23—C18—C4	132.22 (14)
C25—C3—H3	107.4	C19—C18—C4	108.63 (13)
C2—C3—H3	107.4	C20—C19—C18	122.52 (16)
C4—C3—H3	107.4	C20—C19—N2	127.77 (16)
C18—C4—C17	101.45 (12)	C18—C19—N2	109.61 (14)
C18—C4—C3	117.85 (12)	C19—C20—C21	117.63 (17)
C17—C4—C3	108.74 (12)	C19—C20—H20	121.2
C18—C4—C5	115.01 (12)	C21—C20—H20	121.2
C17—C4—C5	110.31 (12)	C22—C21—C20	121.07 (17)
C3—C4—C5	103.43 (12)	C22—C21—H21	119.5
N1—C5—C13	111.32 (12)	C20—C21—H21	119.5
N1—C5—C6	112.02 (12)	C21—C22—C23	120.68 (17)
C13—C5—C6	101.67 (12)	C21—C22—H22	119.7
N1—C5—C4	102.87 (11)	C23—C22—H22	119.7
C13—C5—C4	117.40 (12)	C18—C23—C22	119.14 (16)
C6—C5—C4	111.90 (11)	C18—C23—H23	120.4
O1—C6—C7	126.75 (15)	C22—C23—H23	120.4
O1—C6—C5	124.52 (14)	N2—C24—H24A	109.5
C7—C6—C5	108.34 (12)	N2—C24—H24B	109.5
C8—C7—C12	119.87 (15)	H24A—C24—H24B	109.5
C8—C7—C6	132.72 (15)	N2—C24—H24C	109.5
C12—C7—C6	107.13 (13)	H24A—C24—H24C	109.5
C7—C8—C9	118.06 (16)	H24B—C24—H24C	109.5
C7—C8—H8	121.0	O3—C25—O4	123.53 (17)
C9—C8—H8	121.0	O3—C25—C3	125.34 (17)
C10—C9—C8	122.29 (16)	O4—C25—C3	111.12 (14)
C10—C9—H9	118.9	O4—C26—H26A	109.5
C8—C9—H9	118.9	O4—C26—H26B	109.5
C9—C10—C11	120.90 (16)	H26A—C26—H26B	109.5
C9—C10—H10	119.5	O4—C26—H26C	109.5
C11—C10—H10	119.5	H26A—C26—H26C	109.5
C12—C11—C10	116.09 (15)	H26B—C26—H26C	109.5
			
C1—N1—C2—C3	−174.54 (14)	C7—C12—C13—C5	−0.36 (17)
C5—N1—C2—C3	−44.14 (16)	C11—C12—C13—C5	−175.79 (13)
N1—C2—C3—C25	158.48 (14)	N1—C5—C13—C14	−50.7 (2)
N1—C2—C3—C4	31.78 (17)	C6—C5—C13—C14	−170.18 (17)
C25—C3—C4—C18	−8.3 (2)	C4—C5—C13—C14	67.4 (2)
C2—C3—C4—C18	118.29 (15)	N1—C5—C13—C12	122.71 (13)
C25—C3—C4—C17	106.29 (16)	C6—C5—C13—C12	3.27 (15)
C2—C3—C4—C17	−127.12 (14)	C4—C5—C13—C12	−119.15 (14)
C25—C3—C4—C5	−136.46 (14)	C12—C13—C14—C15	2.0 (2)
C2—C3—C4—C5	−9.87 (16)	C5—C13—C14—C15	174.98 (16)
C2—N1—C5—C13	163.81 (13)	C13—C14—C15—C16	−1.2 (3)
C1—N1—C5—C13	−66.09 (17)	C14—C15—C16—C11	−0.5 (3)
C2—N1—C5—C6	−83.11 (15)	C12—C11—C16—C15	1.3 (2)
C1—N1—C5—C6	47.00 (18)	C10—C11—C16—C15	−176.25 (17)
C2—N1—C5—C4	37.23 (15)	C19—N2—C17—O2	−169.50 (15)
C1—N1—C5—C4	167.34 (13)	C24—N2—C17—O2	1.8 (3)
C18—C4—C5—N1	−145.28 (12)	C19—N2—C17—C4	9.62 (17)
C17—C4—C5—N1	100.76 (14)	C24—N2—C17—C4	−179.04 (16)
C3—C4—C5—N1	−15.38 (14)	C18—C4—C17—O2	169.98 (15)
C18—C4—C5—C13	92.14 (15)	C3—C4—C17—O2	45.1 (2)
C17—C4—C5—C13	−21.82 (18)	C5—C4—C17—O2	−67.68 (19)
C3—C4—C5—C13	−137.96 (13)	C18—C4—C17—N2	−9.13 (15)
C18—C4—C5—C6	−24.86 (17)	C3—C4—C17—N2	−134.01 (13)
C17—C4—C5—C6	−138.82 (13)	C5—C4—C17—N2	113.21 (14)
C3—C4—C5—C6	105.04 (14)	C17—C4—C18—C23	−169.12 (16)
N1—C5—C6—O1	49.29 (19)	C3—C4—C18—C23	−50.6 (2)
C13—C5—C6—O1	168.23 (14)	C5—C4—C18—C23	71.8 (2)
C4—C5—C6—O1	−65.65 (19)	C17—C4—C18—C19	5.61 (15)
N1—C5—C6—C7	−123.94 (13)	C3—C4—C18—C19	124.14 (14)
C13—C5—C6—C7	−5.00 (15)	C5—C4—C18—C19	−113.43 (14)
C4—C5—C6—C7	121.13 (13)	C23—C18—C19—C20	−1.4 (2)
O1—C6—C7—C8	5.6 (3)	C4—C18—C19—C20	−176.94 (15)
C5—C6—C7—C8	178.65 (16)	C23—C18—C19—N2	175.13 (14)
O1—C6—C7—C12	−168.02 (15)	C4—C18—C19—N2	−0.41 (17)
C5—C6—C7—C12	5.01 (16)	C17—N2—C19—C20	170.31 (16)
C12—C7—C8—C9	0.0 (2)	C24—N2—C19—C20	−1.0 (3)
C6—C7—C8—C9	−172.97 (16)	C17—N2—C19—C18	−5.99 (18)
C7—C8—C9—C10	1.7 (3)	C24—N2—C19—C18	−177.26 (16)
C8—C9—C10—C11	−1.4 (3)	C18—C19—C20—C21	1.0 (2)
C9—C10—C11—C12	−0.6 (2)	N2—C19—C20—C21	−174.81 (16)
C9—C10—C11—C16	176.92 (17)	C19—C20—C21—C22	0.1 (3)
C8—C7—C12—C11	−2.1 (2)	C20—C21—C22—C23	−0.8 (3)
C6—C7—C12—C11	172.48 (13)	C19—C18—C23—C22	0.6 (2)
C8—C7—C12—C13	−177.61 (13)	C4—C18—C23—C22	174.91 (16)
C6—C7—C12—C13	−3.00 (17)	C21—C22—C23—C18	0.4 (3)
C10—C11—C12—C7	2.4 (2)	C26—O4—C25—O3	−3.1 (3)
C16—C11—C12—C7	−175.42 (14)	C26—O4—C25—C3	176.22 (17)
C10—C11—C12—C13	177.41 (14)	C2—C3—C25—O3	−7.5 (3)
C16—C11—C12—C13	−0.4 (2)	C4—C3—C25—O3	114.4 (2)
C7—C12—C13—C14	174.18 (14)	C2—C3—C25—O4	173.17 (15)
C11—C12—C13—C14	−1.3 (2)	C4—C3—C25—O4	−64.93 (19)

### Hydrogen-bond geometry (Å, º)

**Table:**

*D*—H···*A*	*D*—H	H···*A*	*D*···*A*	*D*—H···*A*
C10—H10···O2^i^	0.93	2.60	3.268 (2)	130

Symmetry code: (i) *x*, −*y*+3/2, *z*−1/2.
